# Phaeochromocytoma and Acromegaly: a unifying diagnosis

**DOI:** 10.1530/EDM-14-0036

**Published:** 2014-05-01

**Authors:** C Mumby, J R E Davis, J Trouillas, C E Higham

**Affiliations:** Department of EndocrinologyManchester Royal InfirmaryManchesterUK; 1 Department of Histology and Molecular EmbryologyUniversité de LyonLyonFrance; 2 Department of EndocrinologyChristie Hospital NHS Foundation TrustManchesterUK

## Abstract

A 52-year-old lady was referred after a 5 cm left adrenal mass was detected on computed tomography (CT) scanning. She was asymptomatic although was noted to have acromegalic facies. Blood pressure (BP) was normal but plasma normetanephrines were raised to 2.81 mmol/l (<1.09) and urinary normetadrenaline excretion 5.3 μmol/24 h (0–4.3). Adrenal biochemistry screen was otherwise normal. Metaiodobenzylguanidine (MIBG) scan demonstrated uptake in the adrenal lesion. Growth hormone (GH) nadir on oral glucose tolerance test (OGTT) was 2.2 ng/ml with an elevated IGF1 level of 435 ng/ml (72–215), confirming acromegaly biochemically. The remainder of the pituitary screen was normal. A magnetic resonance imaging (MRI) scan of the pituitary revealed an enlarged pituitary gland with a microadenoma/cyst of 2–3 mm in diameter. Alpha blockade was achieved with a titrated dose of phenoxybenzamine before a successful laparoscopic hand-assisted left adrenalectomy. Postoperative biochemical testing revealed a normal plasma normetanephrine level of 0.6 nmol/l (<1.09) and a metanephrine level of 0.35 nmol/l (<0.46 nmol/l). Nadir on OGTT was normal at 0.07 ng/ml with an IGF1 level within the reference range at 111 ng/ml (75–215). Histology demonstrated a well-circumscribed and encapsulated oval mass with microscopic features typical for a phaeochromocytoma. The sections stained strongly positive for GHRH in 20% of cells on immunocytochemistry. Genetic analysis showed no pathogenic mutation. This is a report of the rare condition of a phaeochromocytoma co-secreting GHRH resulting in clinical and biochemical acromegaly. Neuroendocrine tumours can stain positive for GHRH without coexisting acromegaly, but the resolution of patient symptoms and normalisation of serum GH and IGF1 levels following surgery imply that this was functional secretion. Pituitary surgery should be avoided in such cases.

**Learning points:**

- Incidental findings on imaging require thorough investigation to determine the presence of serious pathology.
- Acromegaly and phaeochromocytoma are rarely coincident in the same patient. If this occurs, co-secretion of GHRH from the phaeochromocytoma or the presence of underlying genetic abnormalities must be considered.
- Acromegaly is due to ectopic GHRH-secreting neuroendocrine tumours in <1% of cases, most commonly pancreatic or bronchial lesions.
- Co-secretion of GHRH from a phaeochromocytoma is extremely rare.
- In such cases, the pituitary gland may appear enlarged but pituitary surgery should be avoided and surgical treatment of the neuroendocrine tumour attempted.

## Background

Increasing reliance on radiological investigation and the improvement in imaging resolution have led to a significant number of incidental findings or ‘incidentalomas’ presenting to Endocrine teams for investigation. Studies of patients undergoing abdominal CT scanning have suggested a prevalence of adrenal incidentaloma of 0.4–10% varying with imaging resolution and the age of subjects (1) (2) (3).

This case describes an extremely rare condition initially presenting as the common referral of adrenal incidentaloma. The case highlights the importance of thorough endocrine investigation of incidental findings to allow an accurate diagnosis and appropriate management.

## Case presentation

**Figure 1:**
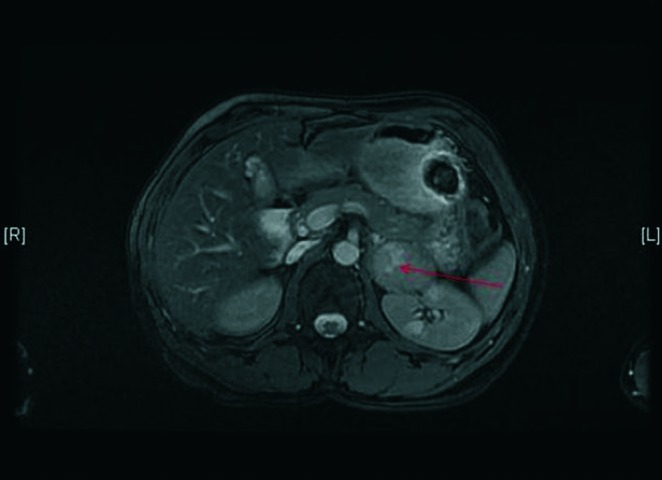
Sagittal T2-weighted MRI of abdomen showing left adrenal mass. (indicated by arrow)

A 52-year-old female was referred to the Endocrine team after an incidental finding of a left adrenal mass (5×4.5×3.5 cm) detected on abdominal CT scan performed for suspected pyelonephritis and renal calculi (Fig. 1).

There was no significant past medical or family history. The history revealed no symptoms other than mild sweating. She was noted to have acromegalic facies on examination. Her blood pressure was normal at 110/60 mmHg rising to a maximum of 135/82 mmHg on 24 h monitoring.

## Investigation

**Figure 2:**
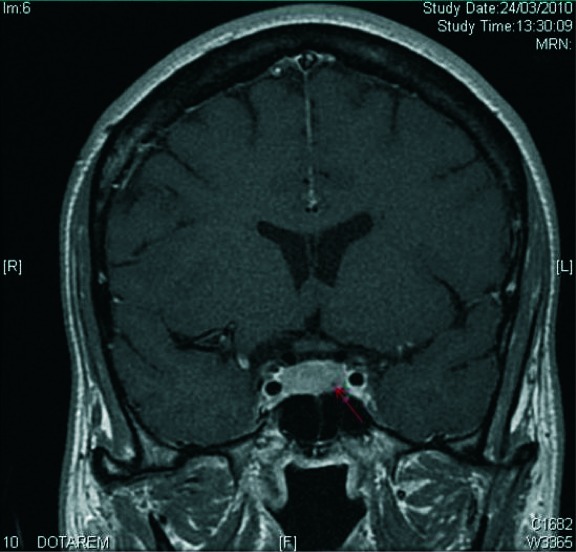
Coronal T1-weighted postcontrast MRI of pituitary showing enlarged pituitary with a small microadenoma or cyst (indicated by arrow).

Biochemical testing revealed raised plasma normetanephrine levels of 2.81 mmol/l (<1.09) and raised urinary normetadrenaline levels of 5.3 μmol/24 h (0–4.3). Aldosterone, renin, adrenal, androgen levels and 24 h urinary free cortisol excretion were normal. Plasma growth hormone (GH) levels failed to suppress adequately during a suppression test with a nadir of 2.2 ng/ml (<0.4 ng/ml). Serum insulin-like growth factor 1 (IGF1) was elevated at 435 ng/ml (75–215). The remainder of the pituitary hormones were within normal limits. Serum corrected calcium was normal. Adrenal magnetic resonance imaging (MRI) and an MIBG scan confirmed a functioning left-sided adrenal lesion. Pituitary MRI revealed a bulky pituitary gland with a small microadenoma or cyst measuring 2–3 mm in diameter (Fig. 2).

## Treatment

This patient received alpha blockade with oral phenoxybenzamine 10 mg daily titrated to 20 mg twice daily. She then underwent a successful laparoscopic hand-assisted left adrenalectomy. Her blood pressure remained stable throughout the procedure.

## Outcomes and follow-up

**Figure 3:**
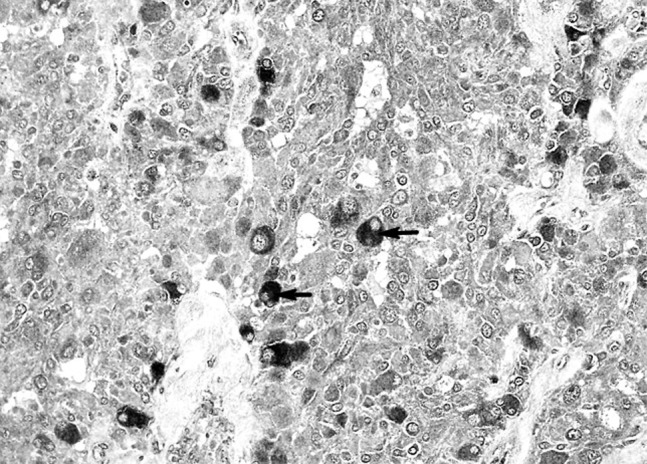
GHRH staining of the phaeochromocytoma. GHRH-strongly positive cells are indicated by arrows.

Postoperative biochemical testing demonstrated normalisation of plasma normetanephrine to 0.6 nmol/l (<1.09) and metanephrine to 0.35 nmol/l (<0.46). During a repeat GH suppression test, GH levels suppressed normally (nadir 0.07 ng/ml) with an IGF1 level in the reference range of 111 ng/ml, confirming successful treatment of the acromegaly. Histology of the adrenal tissue demonstrated a well-circumscribed and encapsulated oval mass with microscopic features typical for a phaeochromocytoma. Immunohistochemistry was performed at the Université de Lyon. Of the total number of cells, 20% showed strongly positive GHRH staining using an anti-GHRH polyclonal antiserum at a dilution of 1/200–1/2000, and 50% were slightly positive (Fig. 3).

Genetic testing has been undertaken with no evidence of pathogenic mutations in *SDHA*, *SDHAF2*, *SDHB*, *SDHC*, *SDHD*, *RET*, *MAX*, *TMEM127* or *VHL* genes.

The patient remains asymptomatic. Biochemical surveillance with plasma metanephrines and IGF1 has been normal, 2 years following surgery. A repeat pituitary MRI 3 years after the initial scan shows a slight reduction in the size of the pituitary overall with no change in the appearance of the microadenoma/cyst.

## Discussion

This is a report of the rare condition of a phaeochromocytoma co-secreting GHRH causing clinical and biochemical acromegaly.

GHRH-secreting neuroendocrine tumours account for <1% of acromegaly, and the most common causative lesions are pancreatic or bronchial carcinoid. To date, there have been <75 cases reported in the literature (4). There are two previous case reports of acromegaly due to co-secretion of GHRH by phaeochromocytoma (5) (6). A recent series of such cases have been reported in France: 21 patients were identified with ectopic secretion of GHRH, 12 cases were secondary to pancreatic lesions and seven bronchial, and there were no phaeochromocytoma cases. Pituitary imaging reported abnormalities in 15 cases. Prognosis appeared good after resection of the lesions with 91% achieving remission and 85% 5-year survival (7).

Acromegaly and phaeochromocytoma rarely occur coincidentally in the same patient. If this occurs, co-secretion of GHRH from the phaeochromocytoma or the presence of underlying genetic abnormalities must be considered. Dénes *et al.* (8) have identified mutations in phaeochromocytoma/paraganglioma genes and pituitary genes in patients and families with co-existing adrenal and pituitary tumours.

Neuroendocrine specimens can stain positively for GHRH though without clinical or biochemical acro-megaly, which may be due to a low concentration or degradation of GHRH in the vesicles or defects in secretion (9). In this case, the resolution of the patient's symptoms and normalisation of serum GH and IGF1 levels following adrenal surgery imply that this was functional secretion. The pituitary may appear enlarged due to hypertrophy, but pituitary surgery should be avoided in such cases with a focus on resection of the ectopic lesion.

## Patient consent

Written informed consent has been obtained from the patient for publication of the case report and the accompanying images.

## Author contribution statement

C Mumby analysed the data and images and prepared the case report under the supervision of C E Higham. J R E Davis is the consultant endocrinologist responsible for the patient. J Trouillas performed histological studies.
